# The Significance of Ventricular Topology in the Analysis of Congenitally Malformed Hearts

**DOI:** 10.3390/jcdd9050155

**Published:** 2022-05-12

**Authors:** Adrian C. Crucean, Diane E. Spicer, Robert H. Anderson

**Affiliations:** 1 Birmingham Women’s and Children’s Hospital, NHS Foundation Trust, Birmingham B4 6NH, UK; sejjran@ucl.ac.uk; 2 Johns Hopkins All Children’s Hospital, St. Petersburg, FL 33701, USA; spicerpath@hotmail.com

**Keywords:** ventricular topology, segmental sequential analysis, segmental connections

## Abstract

There are still confusing descriptions of how congenitally malformed hearts should be categorised, even in their simplest forms. Despite repeated attempts toward a unified and simplified analysis, morphologists and clinicians continue to use different nomenclatures. This variability has a profound impact not only on how we communicate with patients but also on how the healthcare professionals produce clinical reports, research papers and educational and training materials, not to mention the impact on other levels such as managerial, administrative, coding, financial and media communications. Moreover, there are influences on how we actually treat patients based on a different understanding of nomenclature. This paper aims to explain a method of analysing the cardiac segments and their connections based on the current understanding of structural development.

## 1. Introduction

The introduction of segmental analysis by Richard Van Praagh revolutionised the description of the congenitally malformed heart. Prior to the 1960s, complexly malformed hearts would often be placed in a category labelled “miscellaneous”. Van Praagh and his colleagues, in their paradigmatic studies, pointed out that the heart itself could be analysed in terms of its so-called segments [1,2]. The limited variability in each of the segments, and the limited ways in which their cavities could be joined, or not joined, across the atrioventricular and ventriculo-arterial junctions, meant that each individual heart could be analysed and classified in a logical fashion, even if the combination of lesions within the heart had not previously been encountered. The system was first described in the setting of right-sided hearts [1] and then in an analysis of hearts with a double inlet ventricle [2]. In a subsequent review, Van Praagh emphasised the capacity of segmental analysis to cater for all situations [3]. Since we have been set the task of discussing the role of ventricular topology when the heart is congenitally malformed, we should emphasise that the essence of the Van Praaghian approach was the creation of segmental “sets”. Each set established the basic scaffold of the cardiac structures in terms of atrial, ventricular, and arterial “segments”.

To simplify descriptions, codes were identified for each segment, with the segmental sets enclosed in brackets. Thus, the atrial chambers were considered to occupy their usual arrangement, or “solitus”, which was represented by “S”. The mirror-imaged arrangement was said to be inverted, and hence represented by “I”. A third arrangement was considered to be ambiguous and represented by “A”. The key step in the analysis and descriptions of the ventricular segment of the heart was to recognise the direction of ventricular looping. For the segmental sets, Van Praagh and his colleagues described two basic patterns, which were mirror images of each other [1,2,3]. These were initially interpreted in terms of dextro- and levo-bulboventricular looping and were represented in the sets as “D” and “L”. For the arterial segment, the options were the normal, represented as “S”, the mirror-imaged arrangement, or “I”, and two options for an abnormal location of the aortic root, which were coded as “D” and “L”. The segmental arrangement of the normal heart, therefore, was coded as {S,D,S}.

The ventricular loop itself, which is the basis for the understanding of topology, has inlet and outlet components during its development. The origin of these components is from the first and second heart fields, but this feature is of little consideration for the understanding of topology. It is more important to understand the process termed “ballooning”, which produces the apical components of the ventricles [4]. In the setting of rightward looping, the developing right ventricle balloons to the right of its left ventricular counterpart, thus producing the “D” arrangement. With leftward looping, in contrast, the right ventricle is formed to the left of its partner, providing the “L” variant. Van Praagh subsequently pointed out that the two patterns could be analysed on the basis of “handedness”, or ventricular chirality [5]. This approach was based on the relationships of the two ventricles within the overall ventricular mass. It improved on the original approach based on looping itself since the intrinsic relationship of the ventricles within the ventricular mass is not changed by subsequent rotation of the ventricular cone.

Over the same period in which the Van Praaghian system had evolved, however, a group working in Europe, including one of the authors of this review, had suggested that the initial segmental approach could be modified to include details of how the segments themselves were joined together. This variation has subsequently become known as the sequential segmental approach [6]. Those promoting this sequential variation, accepting the notion of handedness as established by Van Praagh [5], argued that the intrinsic arrangement of the components of the morphologically right ventricle could be used to establish the right-handed and left-handed patterns of ventricular topology [6]. It is the importance of the notion of topology as defined by those using the sequential segmental approach that will be the focus of our review. We will show that only by combining the concepts of topology with the fashion in which the atrial and ventricular chambers are joined together is it possible to account for rarer arrangements of congenitally malformed hearts. This information cannot be provided simply by using the “segmental sets”. To provide the background for appreciation of such subtle nuances, we begin our review with a brief account of the evolution of the segmental as opposed to the sequential segmental approaches.

## 2. Segmental versus Sequential Segmental Analysis

As we emphasised in our introduction, within the approach as initially described by Van Praagh [3], the arrangement of the ventricular loop was the middle component of the segmental “sets” that provide the basis for subsequent analysis. Within this system, congruency between the arrangement of the atrial and ventricular segments was defined as representing atrioventricular concordance. This was irrespective of the fashion in which the cavities of the atrial chambers joined, or failed to join, the underlying ventricular cavities. Within the original system, therefore, individuals having the segmental combinations of {S,D,*}, or {I,L,*}, were deemed to have atrioventricular concordance, even in the settings of double inlet ventricle or tricuspid atresia. The asterisk is added in the segmental notation simply because the arrangement of the arterial segment does not change the key relationships between the atrial and ventricular segments. In the situations of double inlet left ventricle or tricuspid atresia, the arrangement itself would first be described, followed by the segmental set, as, for example, with DILV {S,D,D} when there was a usual atrial arrangement, d-looping of the ventricular mass, and the aortic root to the right of the pulmonary root. For those using Van Praaghian nomenclature in an appropriate fashion, therefore, it follows that it is always necessary to provide a full segmental set for each individual. Those with the segmental sets {S,L,*} or {I,D,*}, in which the ventricular loop is incongruent with the atrial arrangement, were described as having atrioventricular discordance, again irrespective of the specific connections of the chambers across the atrioventricular junctions.

The proponents of sequential segmental analysis, when making their own definitions, had not fully understood these initial concepts [6,7]. The emphasis of the sequential approach, whilst recognising the need to begin the analysis with the establishment of the arrangement of the individual segments, was to account for the specific fashion in which the atrial cavities were joined, or not joined, to the ventricular cavities. It was unfortunate, therefore, that in their original account [7], the term “atrioventricular concordance” was used only to account for the arrangement in which atrial cavities were joined with anatomically appropriate ventricular cavities [7]. In retrospect, these arrangements would have been much better described in terms of concordant atrioventricular connections, emphasising, in this way, that the overall approach was based on the union, or non-union, of the cavities of the segments across the junctions between them. In a similar fashion, those promoting sequential analysis initially used “atrioventricular discordance” not to indicate segmental incongruency, but rather to cater only to those individuals in which the atrial chambers were joined in a morphologically inappropriate fashion to the ventricular cavities. Thus, within the sequential approach, individuals with double inlet ventricle or with the variant of tricuspid atresia produced by the absence of an atrioventricular connection were deemed to have specific types of atrioventricular connection, which were discrete from the variants described in terms of atrioventricular concordance or discordance [6,7].

**Figure 1:**
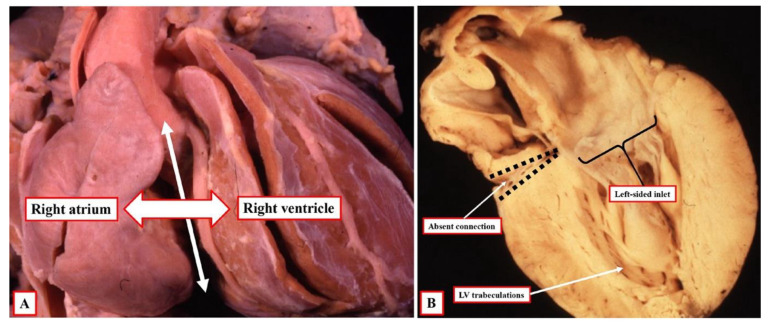
Hearts with the “classical” variant of tricuspid atresia serve to show that “alignments” cannot always be considered to represent the same feature as ‘connections”. As shown in panel (**A**), the right atrium is aligned with the incomplete right ventricle. As shown in panel (**B**), however, the essential feature of the classical variant of tricuspid atresia is the absence of the right atrioventricular connection.

This misinterpretation of the original segmental approach by those promoting sequential analysis then had unfortunate consequences. This was because Van Praagh and his followers argued that the atrial chambers did not “connect” to the ventricles. Instead, the original concept was expanded to introduce the notion of “connecting segments”, which were identified as the atrioventricular canal and the conuses [8]. This then led to a major schism in the description since Van Praagh argued that the segments were “aligned” with each other, rather than being “connected” [9]. This has been an unfortunate development since, in the setting of the commonest variant of tricuspid atresia, the cavity of the right atrium can be aligned with the cavity of the incomplete right ventricle, but in the absence of any direct connection between the cavities of the right atrium and right ventricle (Figure 1). Alignments, therefore, cannot be considered to describe the same features as connections. It is possible, however, that an understanding of the role of ventricular topology can now serve to bridge the schism that has developed between the segmental and sequential segmental approaches. As already emphasised, nonetheless, the initial misinterpretation has been corrected by those promoting the sequential approach. This is because the system now avoids using the terms “atrioventricular concordance” and “discordance”. Instead, when accounting for specific connections across the atrioventricular junctions, those using the sequential approach describe concordant as opposed to discordant atrioventricular connections [10].

## 3. The Development of the Ventricular Mass

**Figure 2:**
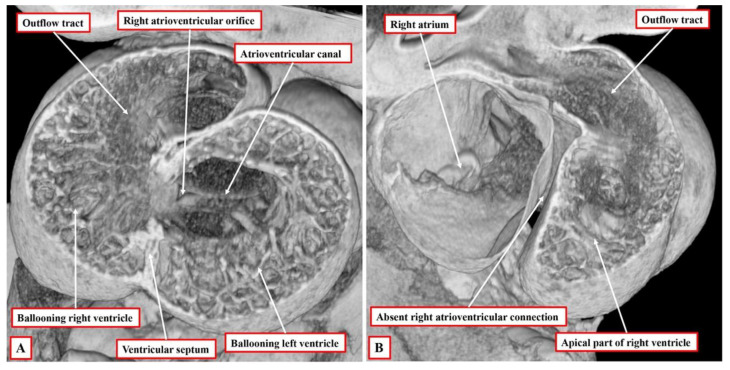
The images show the ventricular loop as seen in a developing murine embryo 10.5 days subsequent to fertilisation. Panel (**A**) shows the frontal section through the loop, while panel (**B**), taken obliquely, reveals that at this early stage, the right atrium has no direct connection with the developing right ventricle.

An understanding of cardiac development now permits us to appreciate the steps involved in the production of the topologic arrangement of the ventricular mass. Topology itself is based on the concept that structures cannot be changed in their make-up simply by the processes of twisting or tilting. If the structure in question is an eccentric three-dimensional entity, which is the case for all of the cardiac segments, then the make-up of the individual segments can only be altered by disassembling their components and then, if this was possible, reconstructing them in an alternative fashion. When first seen, the heart itself is a linear tube. It used to be thought that all the components of the definitive heart were represented in the linear tube. It is now well-established that this is not the case. New material is added, during development, to both the arterial and venous poles of the tube. Its initial part eventually forms little more than the definitive left ventricle. The addition of the new material from the second heart field to form the right ventricle is part of the process of looping, although the full mechanisms underscoring the process have yet to be elucidated [11]. Having looped, the ventricular component of the developing heart initially remains a tube, but with obvious inlet and outlet components. At this initial stage, the inlet part of the loop supports the entirety of the circumference of the atrioventricular canal, whereas the outlet part supports the entirety of the developing outflow tract. Shortly after completion of the process of looping itself, the apical ventricular components can be seen “ballooning” from the outer curvature of the loop, with the left ventricle developing from the inlet part, and the right ventricle developing from the outlet part (Figure 2A).

The arrangement at the early stage is pertinent to the commonest variant of tricuspid atresia, which is shown in Figure 1. Since the right side of the atrioventricular orifice opens into the developing left ventricle, the developing right ventricle lacks a direct atrial input (Figure 2B). In consequence, the developing right ventricle, at this early stage, is incomplete, possessing only apical and outlet components. The left ventricle, in turn, possesses only inlet and apical components. At this early stage, therefore, it is not possible to determine with precision the topological arrangement of the ventricle mass. The relationship of the incomplete right ventricle to the dominant left ventricle could be changed simply by rotating the heart itself.

**Figure 3:**
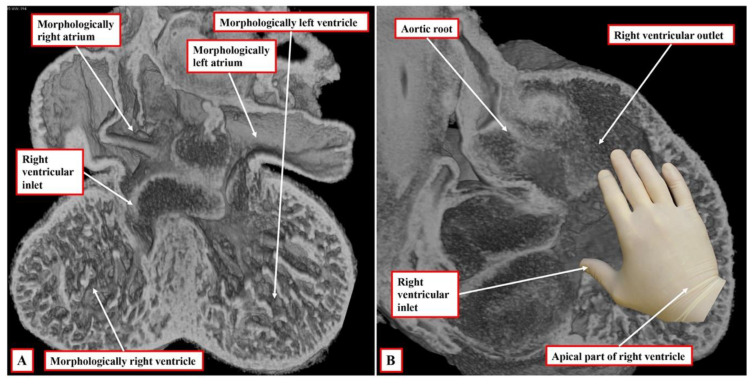
The images are from a developing mouse embryo at embryonic day 11.5. Panel (**A**) is a four-chamber section, showing that, by expansion of the atrioventricular canal, the right ventricle has achieved its own inlet. Panel (**B**) is a long axis section, showing that both arterial trunks still arise from the right ventricle. It can also be seen, however, that because of the formation of the inlet, it is now possible to recognise that the right ventricle shows right-handed topology.

**Figure 4:**
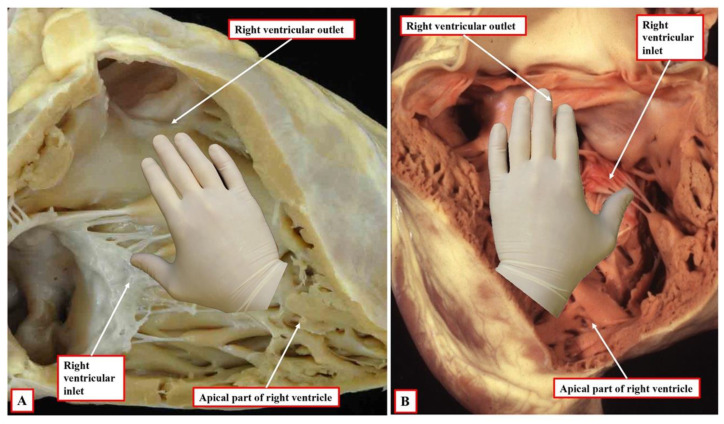
The images show the essence of the patterns of ventricular topology as seen in postnatal hearts. Panel (**A**) shows a normal right ventricle, which accepts on its septal surface only the palmar surface of the right hand when the thumb is placed in the inlet, with the fingers extending into the outlet. Panel (**B**) shows the left-sided morphologically right ventricle from a heart with congenitally corrected transposition. This right ventricle accepts only the palmar surface of the left hand.

It is the next stage of development that sets the scene for the establishment of ventricular topology. Simply by expansion of the atrioventricular canal, the right ventricle achieves its own inlet component. In the murine heart, this process occurs between embryonic days 10.5 and 11.5 (Compare Figure 2 and Figure 3). In the human heart, the period encompasses the fifth and sixth weeks of development subsequent to fertilisation. Once it has achieved its own inlet (Figure 3A), even though it continues to support the entirety of the outflow tract, the right ventricle can be recognised to have achieved right-handed topology (Figure 3B). It is the morphologically right ventricle, by convention, that is used as the arbiter of ventricular topology or chirality. The concept depends on the ability, figuratively speaking, to place the palmar surface of the hands on the septal surface of the right ventricle. The thumb is placed in the tricuspid valve, the wrist occupies the apical trabecular component, and the fingers extend into the ventricular outflow tract. In the normal heart, once the right ventricle has developed its own inlet (Figure 3B), it is only the palm of the right hand that fits the septal surface of the morphologically right ventricle (Figure 4A). In the setting of congenitally corrected transposition, in contrast, which is itself the consequence of the development of discordant atrioventricular connections, and when the atrial chambers are in their usual positions, it is only the palmar surface of the left hand that can be placed on the septal surface (Figure 4B).

It follows, of course, that the left hand could be placed on the septal surface of the morphologically left ventricle in the setting of right-handed topology and the right hand when there is left-handed topology. Convention, nonetheless, has dictated that the right ventricle should be used as the landmark. It also follows that, should congenitally corrected transposition be found in the setting of mirror-imaged atrial arrangement, then the ventricular mass, almost without exception, shows right-handed topology. The caveat, however, is “almost without exception”. On very rare occasions, the ventricular topology is disharmonious with the fashion in which the cavities of the atrial chambers are joined to the ventricles. It is the feature of the flow pathways across the atrioventricular junctions, of course, which is used in the sequential segmental approach to define the segmental connections. Within the segmental approach, however, the so-called “alignments” must be inferred from the information provided within the segmental set. Thus, those with {S,D,*} are presumed to have concordance. This means that, in transposition {S,D,D}, the segmental notation indicates that the atrial and ventricular chambers are joined together in a morphologically appropriate fashion. In transposition {S,L,L}, the segmental notation would indicate that the atrial chambers are joined to morphologically inappropriate ventricles. This system, however, fails to account for disharmony between the morphology of the segments and the connections between them.

## 4. What Is Segmental Disharmony?

**Figure 5:**
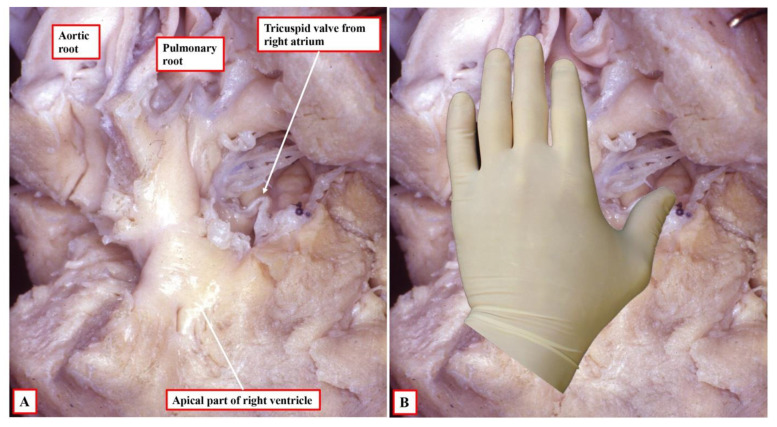
The images show a rare example of segmental disharmony. Panel (**A**) shows the left-sided morphologically right ventricle, which is connected through the tricuspid valve to the right-sided morphologically right atrium; in other words, there are concordant atrioventricular connections. There is also a double outlet from the right ventricle. Panel (**B**) shows that, despite the concordant atrioventricular connections, there is left-handed ventricular topology. This is segmental disharmony.

In very rare circumstances, individuals can be found in whom, when the atrial chambers are in their expected position, and the cavities of the atrial chambers are in continuity with their morphologically appropriate or inappropriate ventricles, the ventricular topology is incongruent with the connections present. As yet, we do not know how, or why, these unusual arrangements might be produced. On occasion, nonetheless, in the presence of usual atrial arrangement and concordant atrioventricular connections, the ventricular topology will be left-handed rather than showing the anticipated right-handed pattern (Figure 5). To emphasise again, such cases are exceedingly rare [12]. As is the case in the heart shown in Figure 5, it is usual to find additional anomalies, such as straddling atrioventricular valves or double outlet right ventricle in these rare examples. For those using the sequential segmental approach, these rare cases represent one of the two occasions when, in addition to describing the atrioventricular connections, it is also necessary specifically to account for the disharmonious ventricular topology. For those using the segmental approach, however, the cases represent a major flaw within the system. It is not possible when using the segmental “sets” to indicate that the so-called “alignments” are disharmonious with the combinations, as coded in the sets themselves.

## 5. When Is It Also Necessary Always to Account for Ventricular Topology?

Disharmony between the segmental arrangements and the connections between them is one of the situations in which description of ventricular topology is mandatory so as to preserve comprehension, but these situations are exceedingly rare. The other situation is encountered with far greater frequency. It is found when, in hearts with isomeric atrial appendages, each atrium is connected to its own ventricle in a biventricular fashion. In the original sequential segmental approach, this arrangement was said to be “ambiguous” [6,7]. In fact, there is no ambiguity. In these settings, half of the atrioventricular junctions are always concordantly connected, while the other half are discordantly connected. The connections, therefore, are properly described as being biventricular and mixed [10]. In such settings, a full description requires that account to be taken, on the one hand, of the type of isomerism, but on the other hand, of the ventricular topology. Thus, the description, for example, of left isomerism with left-handed ventricular topology, is explicit. In these circumstances, it is then also necessary to take account of the venoatrial connections. When the atrial appendages are isomeric, the systemic and pulmonary veins can join the atrial chambers to produce quasi-usual or quasi-mirror-imaged venous drainage. If the venoatrial connections produced quasi-mirror-imaged drainage in the example of left isomerism and left-handed topology described above, it might be presumed, in the setting of concordant ventriculo-arterial connections with parallel arterial trunks, that the atrioventricular connections were also concordant. This might then induce the inference that the atrioventricular node would be in its “regular” position. In reality, in such a setting, because of the left-handed topology and left isomerism, it is very likely that the ventricular conduction axis will arise from an anteriorly located atrioventricular node [13].

## 6. Are Criss-Cross Hearts the Same as Those with Incongruent Segmental Arrangement?

**Figure 6:**
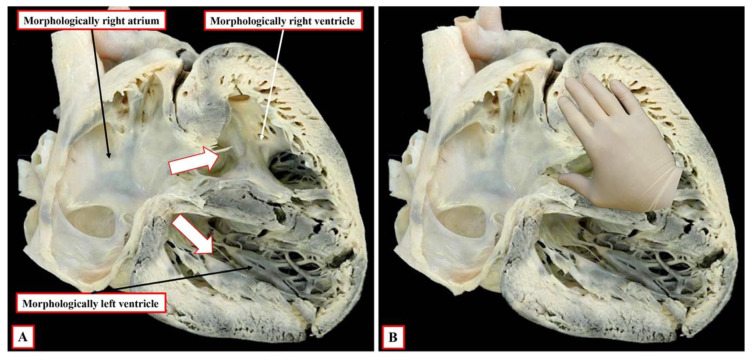
The images show a heart with usual atrial arrangement, concordant atrioventricular connections, with twisting of the atrioventricular connections, as shown in panel (**A**), producing the “cross-cross” arrangement (white arrows with red borders). As shown in panel (**B**), despite the rotation of the heart produced by the twisting, the right ventricle still shows right-handed topology, as expected for hearts with usual atrial arrangement and concordant atrioventricular connections.

As already discussed, the essence of topology is that the arrangement cannot be changed simply by tilting or rotating the object under consideration. The essence of the criss-cross arrangement, however, is that the relationships of the chambers within the heart are changed by the process of rotation [14]. Thus, it is very rare for a criss-cross heart also to show incongruent segmental arrangement. The heart shown in Figure 5 represents one of those rare occurrences when the heart is not only twisted, but the ventricular topology is disharmonious with the atrioventricular connections present. Criss-cross hearts are much more frequent with harmonious atrioventricular segmental arrangements. In these settings, despite the twisting of the ventricular mass along its long axis, the topological arrangement remains as expected for the atrioventricular connections present. The twisting along the long axis takes the morphologically right ventricle into an unexpected position but does not change its topology [15]. In congenitally corrected transposition with the usual atrial arrangement, for example, the morphologically right ventricle is expected to be left-sided. In the criss-cross situation, the twisting moves the right ventricle to a right-sided location. In the heart with usual atrial arrangement and concordant atrioventricular connections, the twisting at the atrioventricular junctions moves the morphologically right ventricle into a left-sided position, but with retention of the anticipated right-handed ventricular topology (Figure 6).

## 7. What about Incongruent Segmental Arrangements at Ventriculo-Arterial Level?

**Figure 7:**
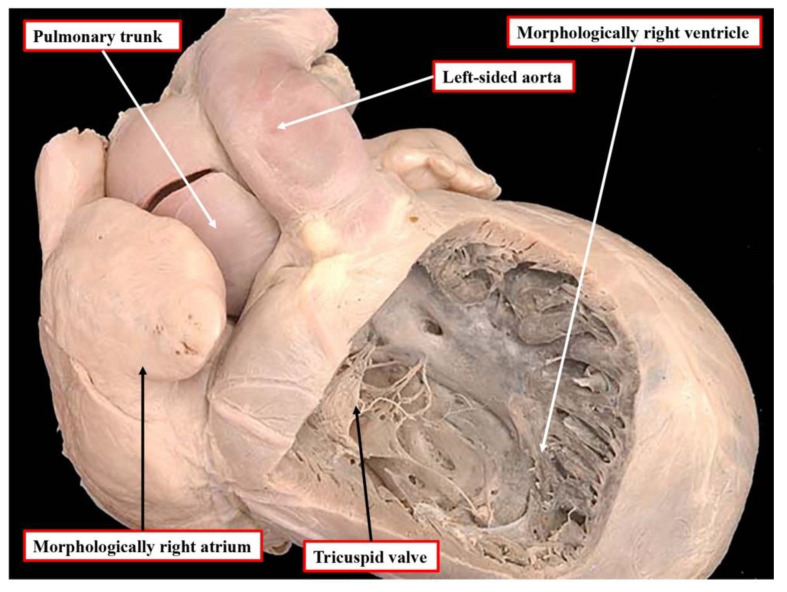
The heart shows an example of transposition with left-sided aorta. This is “l-transposition”, but the heart is not congenitally corrected. There are concordant atrioventricular and discordant ventriculo-arterial connections. This is an example of disharmony between the ventricular and arterial segments of the heart.

When the segmental approach was first introduced, it was not always easy to establish the fashion in which the cavities of the segments were joined together across the atrioventricular and ventriculo-arterial junctions during clinical investigation. In these early days, prior to the introduction of cross-sectional echocardiography, it was conventional to use the “loop rule” to determine the variants of transposition. Thus, in individuals with usual arrangement, the finding of a left-sided aorta arising from the morphologically right ventricle came to be considered an indicator of the presence of congenitally corrected transposition. Indeed, in some centres, even nowadays, “l-transposition” is used as if synonymous with congenitally corrected transposition. The danger of using this approach had already been identified prior to the widespread use of echocardiography. In a seminal study conducted at Great Ormond Street, in collaboration with Dr Van Praagh himself, it was shown that in individuals with regular transposition, in other words, concordant atrioventricular and discordant ventriculo-arterial connections, around one-sixth of those having usual atrial arrangement have left-sided aortas (Figure 7). This investigation, unfortunately, was never published as a full manuscript and is available only in abstract form [16]. For those using the Van Praaghian segmental sets, the segmental disharmony does not create the problems encountered for the atrioventricular junctions. This is because the arrangement, as shown in Figure 7, would be described as transposition {S,D,L}. The inference to be made from the combination of {S,D,*) is that the atrioventricular connections are concordant. The description of transposition indicates that the ventriculo-arterial connections are discordant. Similar ventriculo-arterial disharmony can be found in the setting of the double outlet right ventricle. Thus, in most instances of double outlet right ventricle with usual atrial arrangement and concordant atrioventricular connections, the aortic root is either normally related to the pulmonary trunk or else anterior and rightward. In the past, the anterior location of the aorta would be considered by some as indicative of the presence of “transposition”.

Those using both the segmental and sequential segmental approaches now accept that transposition is synonymous with discordant ventriculo-arterial connections. The finding of an anterior and left-sided aorta can also rarely be found when both arterial trunks arise from the right ventricle when there is usual atrial arrangement and concordant atrioventricular connections. This is another example of disharmony at the ventriculo-arterial junctions. It would properly be described by those using the segmental approach as a double outlet right ventricle {S,D,L}. It follows that it is inappropriate to consider an anterior and left-sided aorta as being indicative of congenitally corrected transposition. Most individuals with congenitally corrected transposition do have left-sided aortas, but not all. Anterior and right-sided aortas are the rule when congenitally corrected transposition is found in individuals with a mirror-imaged atrial arrangement. It also follows, therefore, that “d-transposition” should not be used as a synonym for individuals having concordant atrioventricular and discordant ventriculo-arterial connections.

## 8. Is It Possible to Establish Ventricular Topology in Individuals with Univentricular Atrioventricular Connections and Dominant Left Ventricle?

As we have now demonstrated, the key to establishing ventricular topology is the ability to anchor the thumb in the inlet to the morphologically right ventricle. The essence of hearts having a univentricular atrioventricular connection to a dominant left ventricle is that the right ventricle is incomplete and rudimentary. This is because it lacks its inlet component. Indeed, when there is a double outlet from the dominant left ventricle, on occasion, it can lack both inlet and outlet components. Those using the segmental approach argue that, in the setting of hearts with univentricular atrioventricular connection to a dominant left ventricle, the second chamber present is no more than an infundibulum [17]. There are multiple flaws in the logic underscoring this suggestion, not the least that, if the chamber were truly no more than an infundibulum, it would not be possible to diagnose transposition, which requires the aorta to arise from the morphologically right ventricle or its rudiment. As we have already shown, the evidence is now overwhelming from cardiac development that, from the outset, the small chamber in hearts with a dominant left ventricle possesses an apical trabecular component of right ventricular morphology (Figure 2B).

At the initial stage, however, as also explained, the developing right ventricle lacks its inlet component. It is not possible, therefore, to establish its topology with certainty since it is not possible to anchor the thumb. This remains a problem when seeking to establish topology in postnatal hearts with univentricular atrioventricular connection to a dominant left ventricle, such as double inlet left ventricle or the commonest variant of tricuspid atresia. In most instances, nonetheless, it is possible to infer the likely ventricular topology according to the location of the incomplete ventricle. Almost without exception in these settings, the incomplete right ventricle is carried on the shoulder of the dominant left ventricle. When there is usual atrial arrangement, therefore, a right-sided and anterior location of the incomplete right ventricle can be taken as evidence of right-handed topology, for example, in the heart with tricuspid atresia, as shown in Figure 1A.

**Figure 8:**
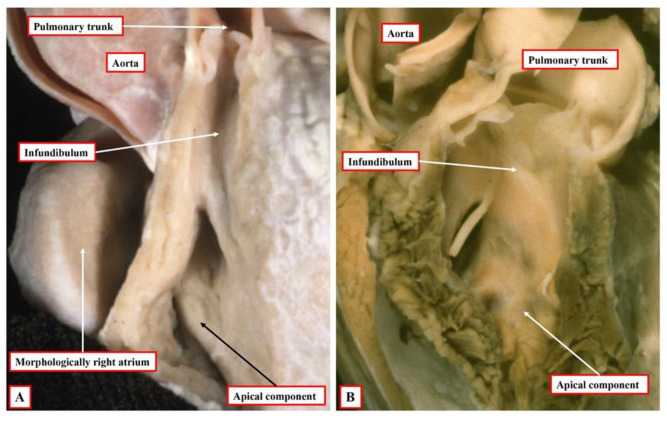
Both of the hearts shown have double inlet left ventricle with incomplete right ventricle and concordant ventriculo-arterial connections. The heart shown in panel (**A**) is a classical “Holmes heart”. The inference can be made that there is a right-handed topology since the incomplete right ventricle is anterior and right-sided relative to the dominant left ventricle. In the heart shown in panel (**B**), the incomplete ventricle is anterior and left-sided. This permits the inference to be made that, during development, there had been leftward ventricular looping. Hence, the incomplete right ventricle can be considered to show left-handed topology. It could, however, be shifted to a right-sided position simply by rotating the heart.

The possibility to make such inferences can also be seen in hearts with a double inlet left ventricle and concordant ventriculo-arterial connections. This lesion is usually known as the “Holmes heart”, with the pulmonary trunk spiralling into the mediastinum having taken origin from an anterior and right-sided incomplete right ventricle (Figure 8A). On occasion, however, the concordantly connected pulmonary trunk can take its origin parallel to an anterior aorta from a left-sided and anterior incomplete right ventricle (Figure 8B).

The inference can be made that, in the “classical” Holmes heart, there is right-handed ventricular topology. In the heart shown in Figure 8B, in contrast, the inference is that, had the tricuspid valve been formed properly, it would have entered the left-sided morphologically right ventricle. Hence, there is left-handed ventricular topology. This finding, however, does create problems for those using segmental notation. This is because the heart shown in Figure 8B would correctly be listed as having a double inlet left ventricle {S,L,D}. This notation, however, could also be correct for hearts having the usual form of double inlet left ventricle with discordant ventriculo-arterial connections, but with a right-sided aorta arising from the left-sided incomplete right ventricle. For those using the segmental approach, therefore, it would also be necessary to arbitrate for the concordant ventriculo-arterial connections in the heart shown in Figure 8B. Those using the sequential approach do not encounter this problem since the description of double inlet left ventricle with left-sided incomplete ventricle and concordant ventriculo-arterial connections is explicit.

## 9. Are There Connecting Segments, and If So, What Happens to Them?

As discussed, it was the suggestion that additional segments were needed to “connect” the major segments that led to the introduction of “alignments” to replace the alternative term “connections”. When the analysis is confined to the developing heart, then it is true that additional components interpose between the atrial chambers and the ventricular loop and between the ventricular loop and the aortic sac. With ongoing development, however, the embryonic atrioventricular canal becomes sequestrated on the atrial side of the plane of atrioventricular insulation, forming the atrial vestibules. The proximal component of the outflow tract then becomes incorporated as the ventricular outflow tracts, with the remainder of the outflow tract transforming into the arterial roots and the intrapericardial arterial trunks. In the postnatal heart, therefore, it is possible to define the ventricular mass as extending from the atrioventricular to the ventriculo-arterial junctions, with no intervening “connecting segments”. Description of the ventricles themselves as possessing inlet, apical, and outlet components then further simplifies the analysis. Moreover, as explained, it is the tripartite ventricular make-up that underscores the establishment of ventricular topology.

## 10. Conclusions

The essential component of the analysis of the ventricular segment of the heart in the process of segmental analysis was the distinction of d- and l-looping. These arrangements were coded as “D” and “L” in the segmental “sets” that continue to be the basis for Van Praaghian descriptions. Those using both a segmental analysis, and its extension into sequential segmental analysis, now recognise that the two variants of looping can also be well described in terms of ventricular topology. For those using the segmental approach, the determination of the topological arrangement then permits inferences to be made with regard to the so-called “alignments” present. This is not necessary for those using sequential analysis since the system depends on specific recognition of the fashion in which the cavities of the segments are connected together. For those using the sequential segmental approach, it is necessary also to account for the topology only when biventricular atrioventricular connections are found in the setting of isomeric atrial appendages and in very rare circumstances when the topology is disharmonious with the connections present.

## Data Availability

The published images have been cleared for publication for educational and research purposes in the public domain.
